# Tissue-Specific Expression of Circ_015343 and Its Inhibitory Effect on Mammary Epithelial Cells in Sheep

**DOI:** 10.3389/fvets.2022.919162

**Published:** 2022-06-28

**Authors:** Xinmiao Wu, Huimin Zhen, Yan Liu, Lu Li, Yuzhu Luo, Xiu Liu, Shaobin Li, Zhiyun Hao, Mingna Li, Liyan Hu, Lirong Qiao, Jiqing Wang

**Affiliations:** Gansu Key Laboratory of Herbivorous Animal Biotechnology, College of Animal Science and Technology, Gansu Agricultural University, Lanzhou, China

**Keywords:** circ_015343, mammary epithelial cells, proliferation, viability, tissue expression, sheep

## Abstract

Circular RNAs (circRNAs) are a kind of non-coding RNA that have an important molecular function in mammary gland development and lactation of mammals. In our previous study, circ_015343 was found to be highly expressed in the ovine mammary gland tissue at the peak-lactation period by using RNA sequencing (RNA-seq). In the present study, the authenticity of circ_015343 was confirmed by using reverse transcriptase-polymerase chain reaction (RT-PCR) analysis and Sanger sequencing. The circ_015343 was derived from the complete 10 exons of aminoadipic semialdehyde synthase (*AASS*), ranging from exon 2 to exon 11 and mainly located in cytoplasm of ovine mammary epithelial cells. The circRNA was found to be expressed in eight ovine tissues, with the highest expression level in the mammary gland and the least expression in *Longissimus dorsi* muscle. The circ_015343 had a lower level of expression in a sheep breed with higher milk yield and milk fat content. The disturbed circ_015343 increased the viability and proliferation of the ovine mammary epithelial cells. The inhibition of circ_015343 also increased the expression levels of three milk fat synthesis marker genes: acetyl-coenzyme A carboxylase alpha (*ACACA*), fatty acid-binding protein 4 (*FABP4*), and sterol regulatory element-binding protein 1 (*SREBP1*), as well as three proliferation-related genes: cyclin dependent kinase 2 (*CDK2*), cyclin dependent kinase 4 (*CDK4*) and proliferating cell nuclear antigen (*PCNA*), but decreased the expression level of its parent gene *AASS*. A circRNA-miRNA-mRNA interaction network showed that circ_015343 would bind some microRNAs (miRNAs) to regulate the expression of functional genes related to the development of mammary gland and lactation. This study contributes to a better understanding of the roles of circ_015343 in the mammary gland of sheep.

## Introduction

The lactation performance of ewes directly affects the survival rate, growth rate and development of lambs before weaning (1). In other words, low milk yield of ewes increases mortality of lambs in early growth stages, especially for multiple-born lambs (2). In this context, the improvement of lactation performance of ewes is of great significance in sheep production. Milk is originated from mammary epithelial cells in mammals, which undergo a series of functional differentiation and become ready for subsequent lactation in pregnancy. The number and activity of mammary epithelial cells influence milk yield and milk composition (3). It is well-known that lactation performance is not only affected by age, parity, nutrition, and lactation stage but also regulated by mRNAs and non-coding RNAs (4, 5). It was therefore possible for improving milk performance by regulating the expression of RNAs in domestic animals.

Circular RNA (circRNA) is a class of non-coding RNA produced by back-splicing of linear mRNAs (6). The circRNAs play important biological functions in cell morphogenesis and life processes in different ways. Firstly, circRNAs existing in the cytoplasm can act as microRNA (miRNA) sponges to increase the target mRNAs expression interfered by the miRNAs (7), and can also interact with RNA binding proteins to translate proteins (8). Additionally, circRNAs existing in the nucleus can regulate gene transcription (9). To date the effect of individual circRNA on mammary gland development and lactation performance of domestic animals have mainly been focused on dairy goats. For example, circ_016910 promoted the proliferation of goat mammary epithelial cells, and the secretion of β-casein and triglycerides (10). The circ_8220 promoted milk synthesis and activity of goat mammary epithelial cells (11). There are some studies that have described the expression profiles of circRNAs in the mammary gland tissue of sheep, but these studies just screened differentially expressed circRNAs between the samples with different genetic backgrounds or with different development periods in the mammary gland tissue. Hao et al. (5) identified 33 differentially expressed circRNAs in the mammary gland between Small Tail Han ewes and Gansu Alpine Merino ewes, and the target genes of some circRNAs were related to mammogenesis and lactation (5). RNA sequencing (RNA-seq) approach was applied to compare circRNA expression levels at peak-lactation with those at the non-lactating period in the mammary gland tissue of Small Tail Han ewes (12). However, little is known about the effect of individual circRNA on mammary gland development in sheep.

In our previous research, circ_015343 was found to be a highly expressed circRNA in the mammary gland tissue at the peak-lactation period in sheep by using RNA-Seq (5), suggesting that circ_015343 may be important for lactation. However, to our knowledge, there have been no reports on the tissue expression of circ_015343 in sheep and its effect on ovine mammary epithelial cells. Accordingly, in this study, we verified the authenticity of circ_015343 and investigated the expression profiles of circ_015343 and its parent gene aminoadipic semialdehyde synthase (*AASS*) in ovine eight tissues, including mammary gland tissue, from the two sheep breeds with different lactation performance. We also analyzed the effect of circ_015343 on the expression of functional genes and the viability and proliferation of ovine mammary epithelial cells.

## Materials and Methods

### Sample Collection and RNA Extraction

The experiments on the sheep were approved by the Animal Experiment Ethics committee of Gansu Agricultural University (Lanzhou, China) with a number of GSAU-ETH-AST-2021-027.

Under the same feeding and management conditions of the Jinzihe Sheep Breeding Company (Tianzhu County, China), three Small Tail Han sheep and three Gansu Alpine Merino sheep at peak lactation (22 days postpartum) were selected and then slaughtered. These ewes were all healthy, 3-year-old and fourth-parity, and the Small Tail Han ewes had higher milk yield and contents of milk fat and protein than Gansu Alpine Merino ewes (5). Their mammary gland, heart, liver, spleen, lungs, kidney, ovary, and *Longissimus dorsi* muscle tissues were collected, and then immediately frozen in liquid nitrogen used for RNA extraction. Meanwhile, a part of the parenchyma of the mammary gland was also collected for culturing ovine mammary epithelial cells.

Total RNA was isolated and purified by using Trizol Reagent (Invitrogen, Carlsbad, CA, USA). The quality and concentration of the RNA were detected by using a NanoDrop 2000 (Thermo Scientific, Woltham, MA, USA) and RNase-free agarose gel electrophoresis. Qualified RNA was reverse transcribed to generate cDNA with Super Script^TM^ II reverse transcriptase (Invitrogen, Carlsbad, CA, USA).

### Authenticity Verification of Circ_015343

Reverse transcriptase-polymerase chain reaction (RT-PCR) and Sanger sequencing were used to validate the presence of circ_015343. Briefly, a divergent primer of circ_015343 that was designed by using primer V3.0 (Table 1) was used to amplify the cDNA. The PCR amplicons were checked by electrophoresis in 1.5% agarose gels and then sequenced by using Sanger sequencing. The comparison of sequences obtained by Sanger sequencing with those from RNA-Seq data and sheep reference genome Oar_rambouillet_v1.0 was performed to validate the presence of the head-to-tail splice junction of circ_015343 by using MEGA V7.0.

**Table 1:** The information of PCR primers.

**Name**	**Forward (5**^′^→**3^**′**^)**	**Reverse (5**^′^→**3^**′**^)**	**Amplicon size (bp)**
Circ_015343	CATTGACAATTTGCCAGCAC	TTCACATCCTCCCGCCTC	198
*AASS*	TCACAGGGACTGGTAATG	AAGATGATGATGGCGACT	169
*β-actin*	AGCCTTCCTTCCTGGGCATGGA	GGACAGCACCGTGTTGGCGTAGA	113
*FABP4*	TGTCCTCAAATTGGCCAGG	AGCAGTGACCGTTCATGAC	188
*ACACA*	GTCCTCTGCCAGTTTCCC	TCCATCACCACAGCCTTC	173
*SREBP1*	CCTCTGTCTCTCCTGCAACC	CCGAGTGACTGGTTCTCCAT	235
*CDK2*	CATGGATGCCTCTGCACTCACTGGC	CTGGCTAGTCCGAAGTCTGCTA	180
*CDK4*	TGGCTACCTCTCGATACGAGCCAGT	CCCGAACGGTGCTGATGG	165
*PCNA*	TCAAGTGGCGTGAACCTACA	TACTAGTGCCAAGGTGTCCG	213
*GAPDH*	ATCTCGCTCCTGGAAGATG	TCGGAGTGAACGGATTCG	227
*U6*	CTCGCTTCGGCAGCACA	AACGCTTCACGAATTTGCGT	94

### Tissue Expression Profiles of Circ_015343 and *AASS*

The RT-qPCR was conducted in triplicate by using the 2 × ChamQ SYBR qPCR Master Mix (Vazyme, Nanjing, China). The gene β*-actin* was used as an internal reference for standardization as suggested by Hao et al. (5). The primer information used for RT-qPCR is shown in Table 1. A 2^−ΔΔCt^ method (13) was used to calculate the relative expression levels of circ_015343 and its parent gene *AASS*.

### Cellular Localization of Circ_015343 and Transfection of Ovine Mammary Epithelial Cells

Ovine mammary epithelial cells were cultured according to the method established by Anand et al. (14). Nuclear and cytoplasmic RNA was separated by using the cytoplasmic nucleus separation kit Minute ^TM^ (Invent Biotechnologies, MN, USA). *U6* and *GAPDH* were chosen as internal controls to calculate the relative expression of circ_015343 in the cytoplasm and nucleus, and the percentage of the circRNA in ovine mammary epithelial cells was accordingly calculated.

The small interfering RNA of circ_015343 (named si-circ_015343) was synthesized in Ribobio company (Ribobio, Guangzhou, China) with a sequence of 5′-GAAGAAATGTCATCAGGTT-3′. The negative control (NC) was also synthesized in the Ribobio company. The ovine mammary epithelial cells were cultured in 12-well plates by using a whole culture medium (DMEM/F12 medium, 10% fetal bovine serum, 100 U/ml penicillin, and streptomycin).

When the density of every well was 80–90%, si-circ_015343 and si-circ_015343 NC were respectively transfected into ovine mammary epithelial cells by using INVI DNA&RNA Transfection Reagent™ (Invigentech, CA, USA). After transfection for 48 h, the transfection efficiency of si-circ_015343 was detected by using RT-qPCR. Meanwhile, the expression levels of three milk fat synthesis marker genes: acetyl-coenzyme A carboxylase alpha (*ACACA*), fatty acid-binding protein 4 (*FABP4*), and sterol regulatory element-binding protein 1 (*SREBP1*), the parent gene *AASS*, as well as three proliferation-related genes: cyclin dependent kinase 2 (*CDK2*), cyclin dependent kinase 4 (*CDK4*) and proliferating cell nuclear antigen (*PCNA*) were detected by using RT-qPCR. has been shown to stimulateTheir primer sequences are listed in Table 1 and *β-actin* was used as an internal reference.

### Effect of Si-Circ_015343 on Viability and Proliferation of Ovine Mammary Epithelial Cells

The cultured ovine mammary epithelial cells were inoculated into 96-well plates containing 100 μl of whole culture medium. After transfection of si-circ_015343 and si-circ_015343 NC into ovine mammary epithelial cells for 42 h, 10 μl of CCK-8 solution was added to each well, and the culture was continued in a 37°C incubator for 2 h. The absorbance of the cell was detected at 450 nm by using a microplate reader (Thermo Scientific, Waltham, MA, USA). Three technical repetitions were performed and cell viability was calculated accordingly.

The ovine mammary epithelial cells were cultured in 24-well plates containing 500 μl of whole culture medium. After transfection for 44 h, 100 ml 50 mM Cell-Light™ EdU reagent (Beyotime, Shanghai, China) was added to each well. The Edu staining result was observed by using a fluorescence microscope IX73 (Olympus, Tokyo, Japan). Five different images were randomly selected for each observation field, and the number of Edu-labeled proliferated ovine mammary epithelial cells was counted by using Image pro-plus V6.0.

### Construction of a CircRNA-MiRNA-MRNA Regulatory Network

The miRanda V3.3a (15) was used to predict the miRNA binding site of circ_015343, as well as the target mRNAs of the miRNAs predicted. A circRNA-miRNA-mRNA regulatory network was constructed by using StarBase V3.0 (16) and then drawn using Cytoscape V3.5.1 (17).

### Statistical Analysis

All analyses were performed by using one-way ANOVA or two-tailed Student's *t*-test in SPSS V22.0 (IBM, NY, USA).

## Results

### Authenticity Verification and Cellular Localization of Circ_015343

**Figure 1:**
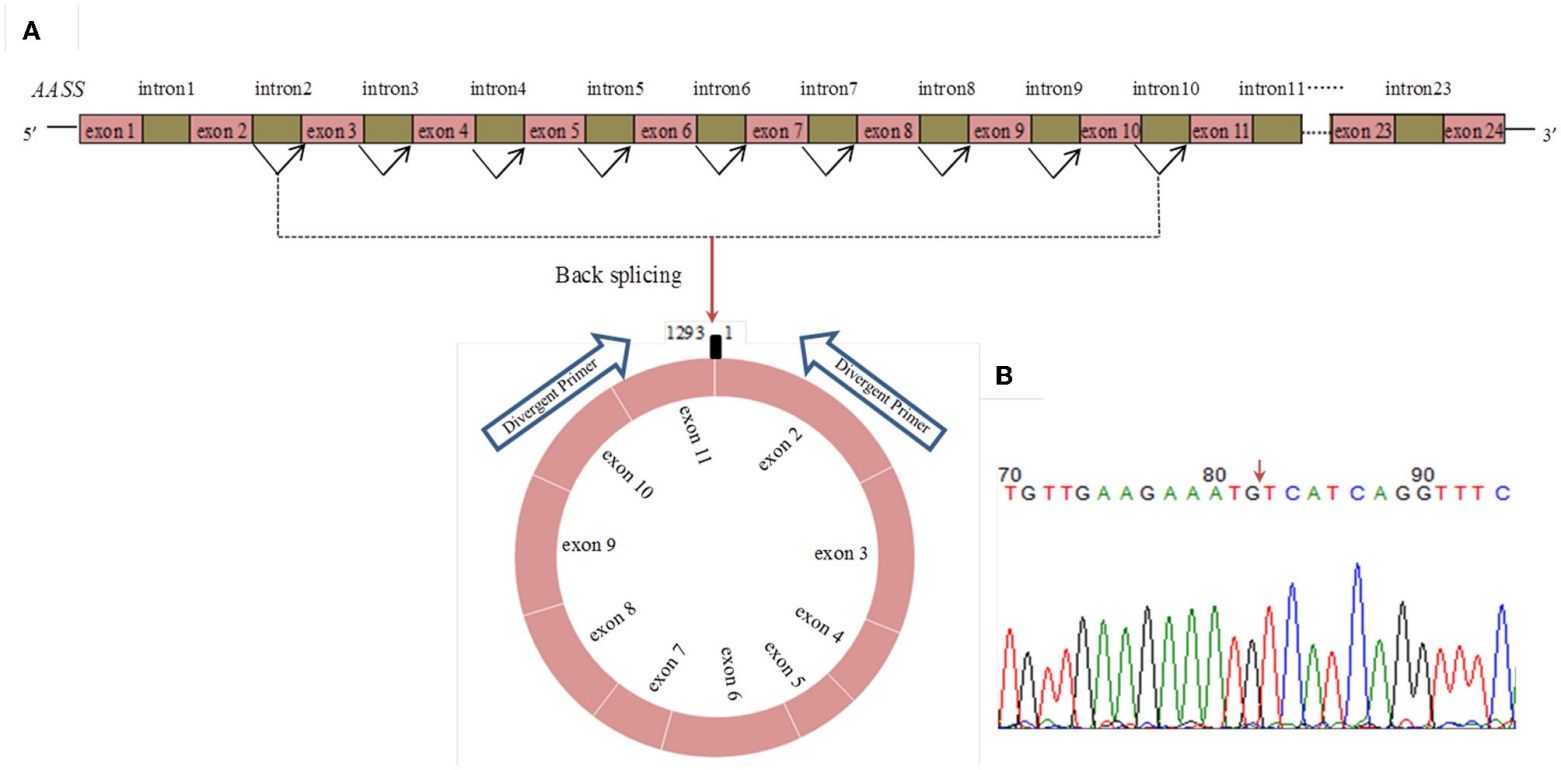
The genomic loci of circ_015343 derived from *AASS* **(A)** and verification of head-to-tail splice junction of circ_015343 by using Sanger sequencing **(B)**. A red arrow indicates the head-to-tail splice junction of circ_015343.

**Figure 2:**
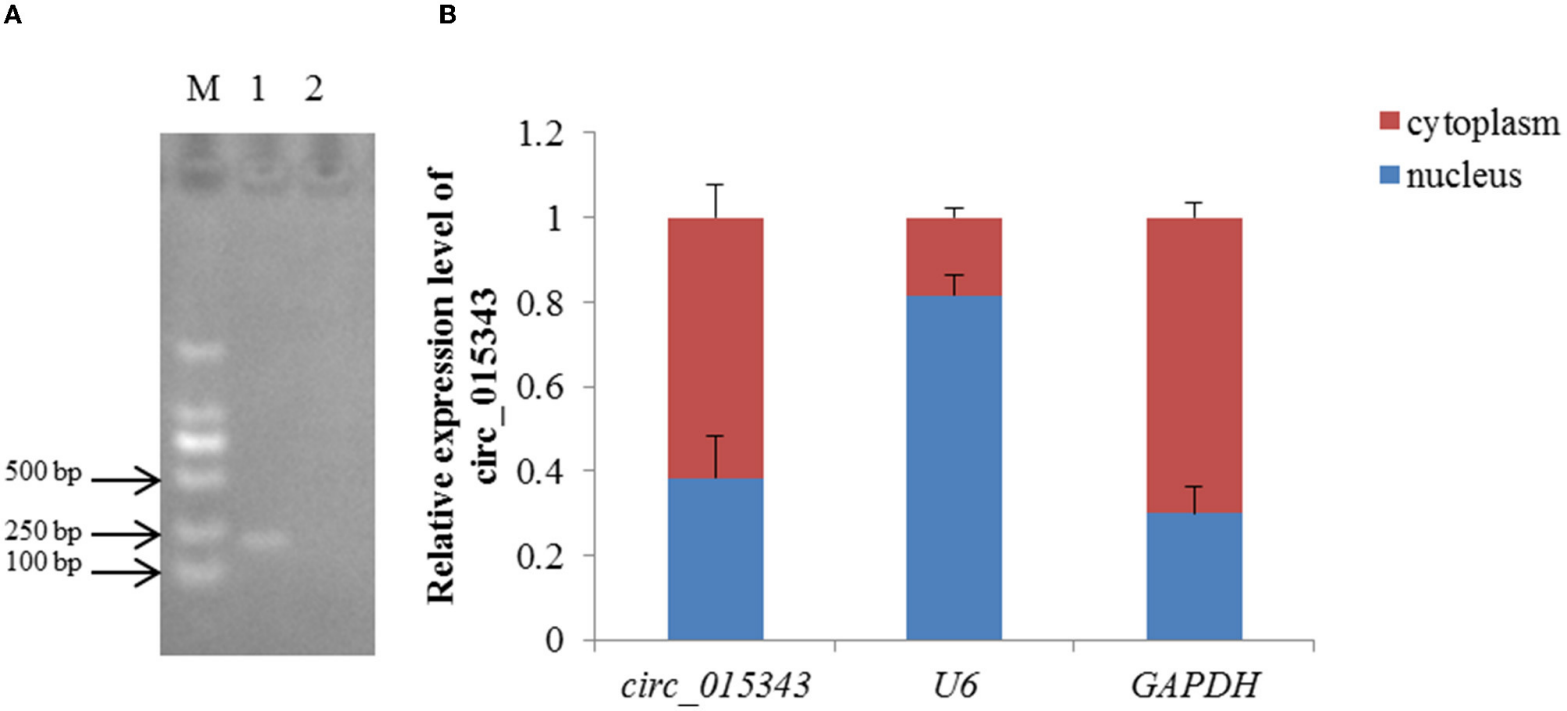
Subcellular localization of circ_015343. **(A)** The RT-PCR expression of circ_015343 in cytoplasm and nucleus of ovine mammary epithelial cells detected by using agarose gel elecrophoresis. M: marker. Numbers above lane indicates the components of ovine mammary epithelial cells studied: 1: cytoplasm; 2: nucleus. **(B)** The RT-qPCR expression level of circ_015343 in cytoplasm and nucleus of ovine mammary epithelial cells of which was separated by cytoplasmic nucleus separation kit. The genes *U6* and *GAPDH* were used as internal references.

RNA-Seq data showed that circ_015343 was an annot_exon circRNA located on ovine chromosome 4 and originated from *AASS* (oar4: 86579656-86598085). The sequence comparison of circ_015343 with *AASS* indicated that circ_015343 was derived from the complete 10 exons of *AASS*, ranging from exon 2 to exon 11 (Figure 1A). The genomic sequence of circ_015343 is 1,293 nucleotides in length. Sanger sequencing results further confirmed the presence of head-to-tail splice junction of circ_015343 (Figure 1B) as suggested by the RNA-Seq analysis, suggesting the authenticity of the circ_015343. RT-PCR analysis revealed that circ_015343 was predominantly expressed in the cytoplasm, but was only weakly expressed or did not express in the nucleus of ovine mammary epithelial cells (Figure 2A). The relative expression levels of circ_015343 in the cytoplasm and nucleus were further detected by using RT-qPCR, and the percentage of the circRNA in ovine mammary epithelial cells was accordingly calculated. The result showed that 62% of circ_015343 is located in the cytoplasm, while 38% is expressed in the nucleus (Figure 2B).

### Expression Profiles of Circ_015343 and *AASS* in Ovine Different Tissues

**Figure 3:**
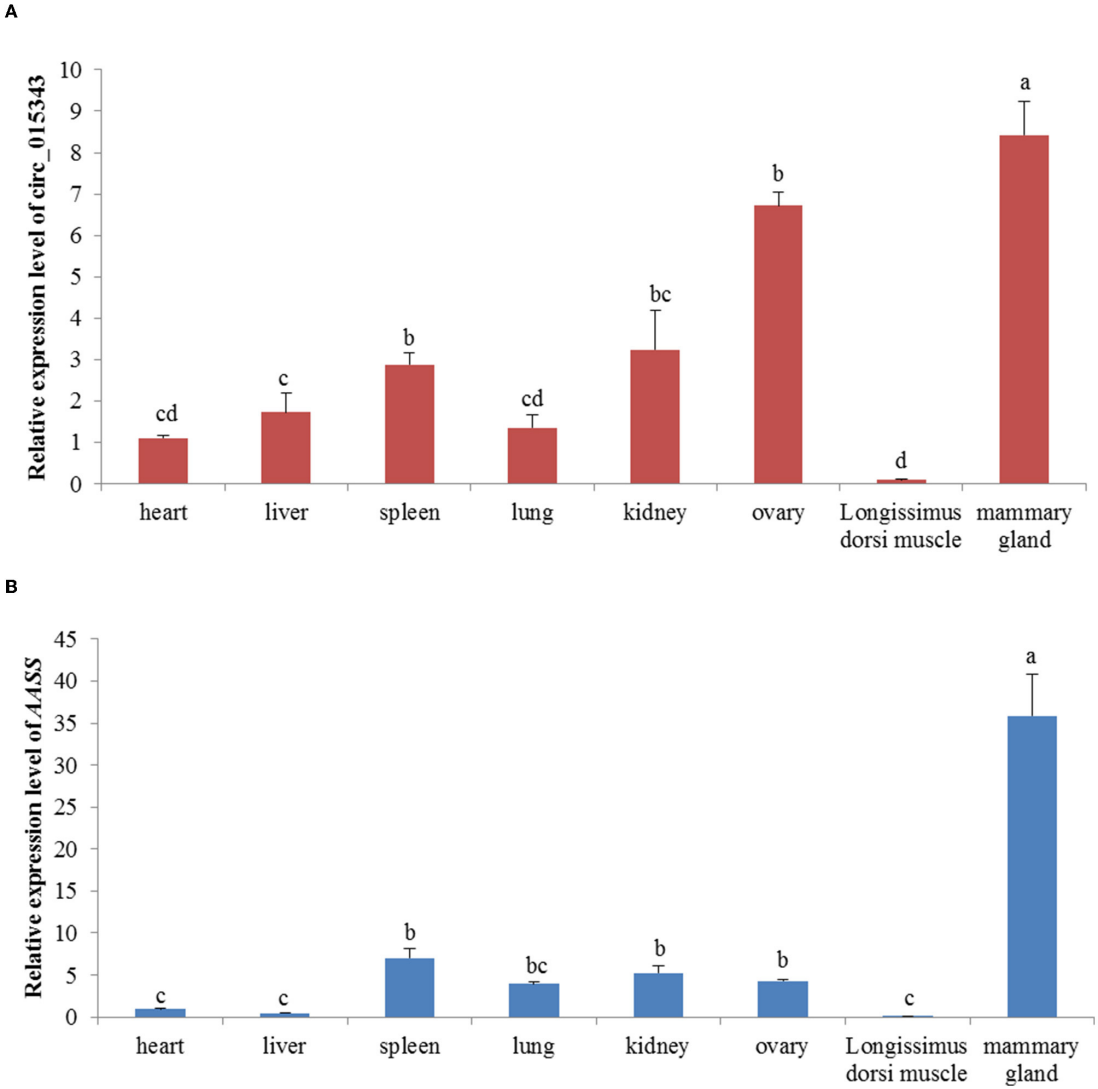
Relative expression of circ_015343 **(A)** and *AASS* **(B)** in ovine eight different tissues. Different lowercase letters above the bars represent significant differences (*p* < 0.05) between tissues.

RT-qPCR results showed that circ_015343 and *AASS* were all expressed in ovine 8 tissues. Ovine circ_015343 had the highest expression levels in the mammary gland and had a relatively high level of expression in the ovary, spleen, and kidney. It was expressed lower in lung, heart, liver, and *Longissimus dorsi* muscle tissues (*p* < 0.05) (Figure 3A). *AASS* exhibited a similar expression tendency with circ_015343, with the highest expression level in the mammary gland tissue and the least expression in heart, liver, and *Longissimus dorsi* muscle tissues (*p* < 0.05) (Figure 3B). These reflect tissue-specific expression patterns of circ_015343 and *AASS* in sheep.

### Expression Levels of Circ_015343 and *AASS* in the Mammary Gland Tissue of the Two Sheep Breeds

**Figure 4:**
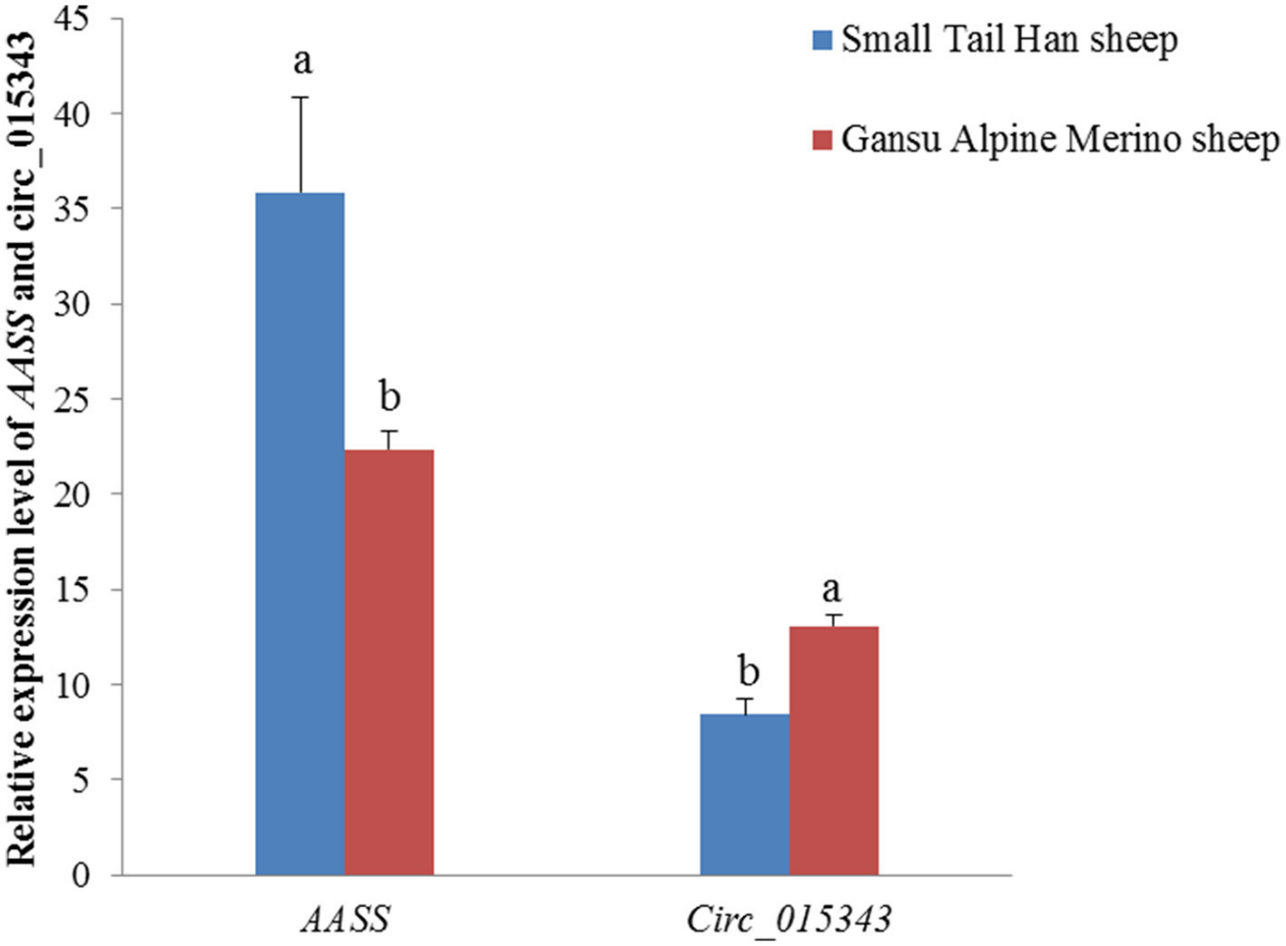
Relative expression levels of *AASS* and circ_015343 in the mammary gland tissue at peak-lactation period in Small Tail Han sheep and Gansu Alpine Merino sheep. Different lowercase letters above the bars represent significant differences (*p* < 0.05) between the two breeds.

In the mammary gland tissue at peak-lactation period, circ_015343 had a relatively lower level of expression (*p* = 0.003), while *AASS* had a higher expression level in Small Tail Han sheep compared to Gansu Alpine Merino sheep (*p* = 0.019) (Figure 4).

### Effect of Circ_015343 on the Expression Levels of Functional Genes

**Figure 5:**
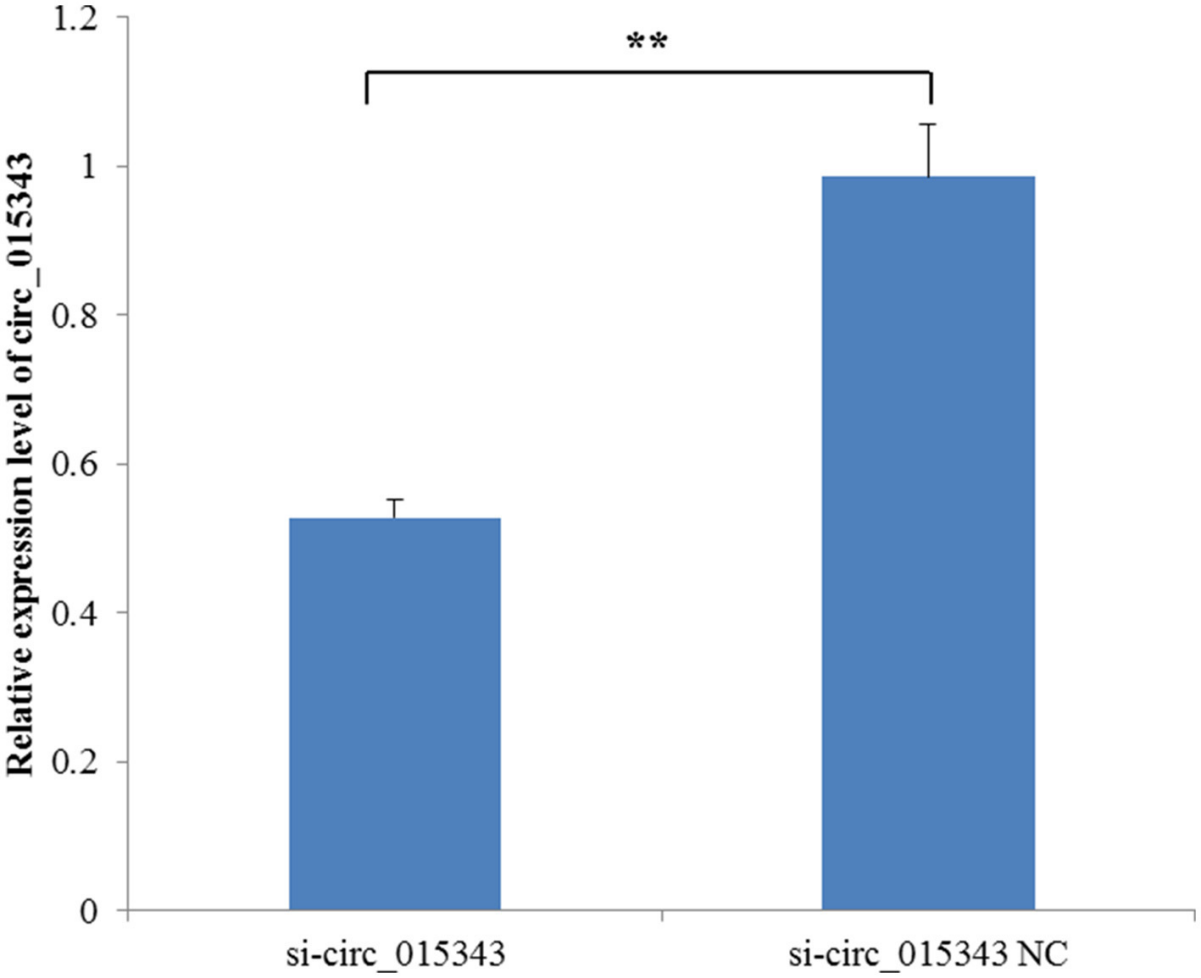
The expression level of circ_015343 when si-circ_015343 and si-circ_015343 NC were transfected into ovine mammary gland epithelial cells. **p < 0.01.

When si-circ_015343 and si-circ_015343 NC were transfected into ovine mammary epithelial cells, RT-qPCR detection results showed that si-circ_015343 significantly decreased the expression level of circ_015343 when compared to si-circ_015343 NC (*p* = 0.001) (Figure 5). This suggests that si-circ_015343 was successfully transfected into ovine mammary epithelial cells.

**Figure 6:**
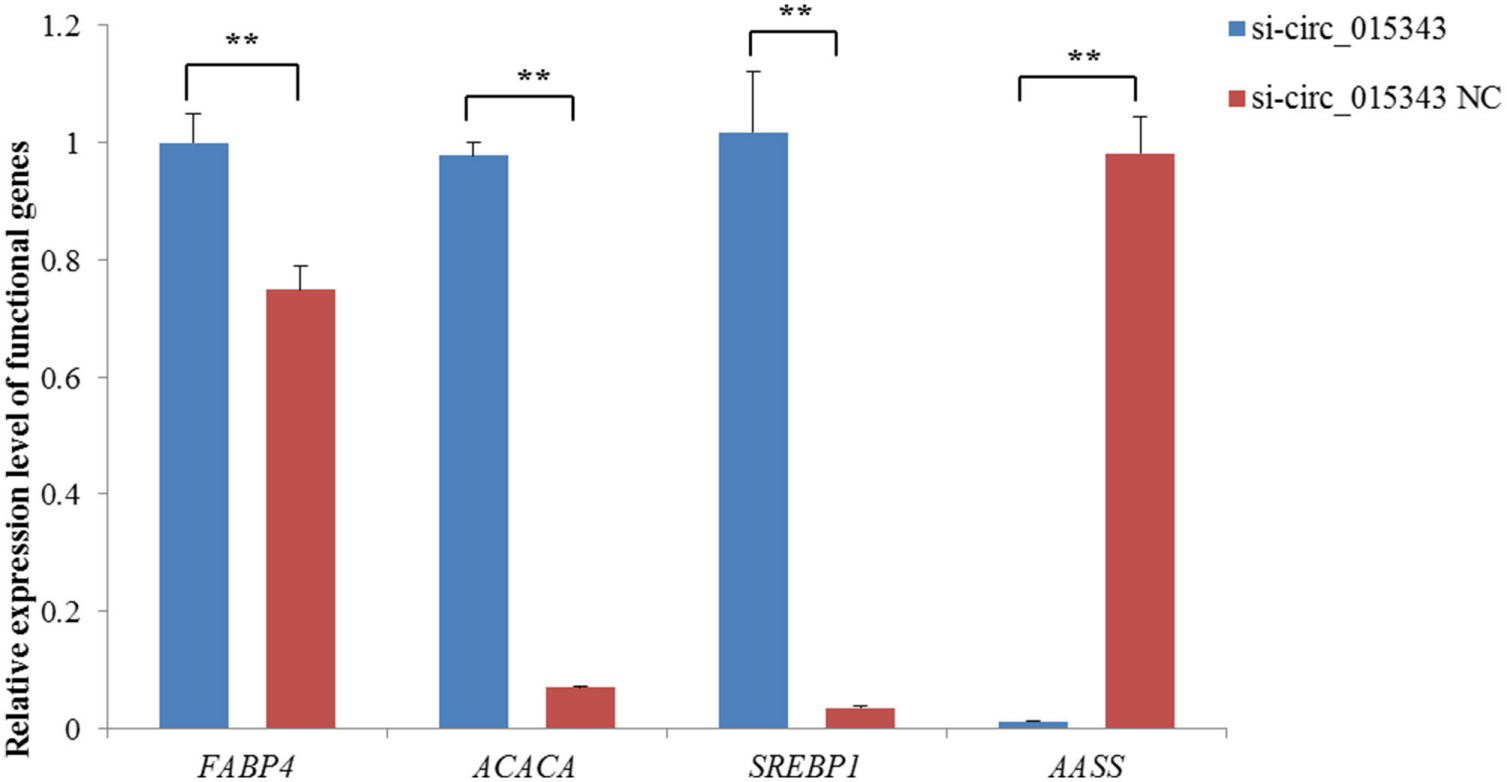
Relative expression levels of functional genes when si-circ_015343 was transfected into ovine mammary epithelial cells. **p < 0.01.

The RT-qPCR results showed si-circ_015343 inhibited the expression of *AASS* (*p* = 0.000), while increased the expression levels of *FABP4* (*p* = 0.005), *ACACA* (*p* = 0.000) and *SREBP1* (*p* = 0.000) when compared to NC group (Figure 6).

### Effect of Circ_015343 on the Viability and Proliferation of Ovine Mammary Epithelial Cells

**Figure 7:**
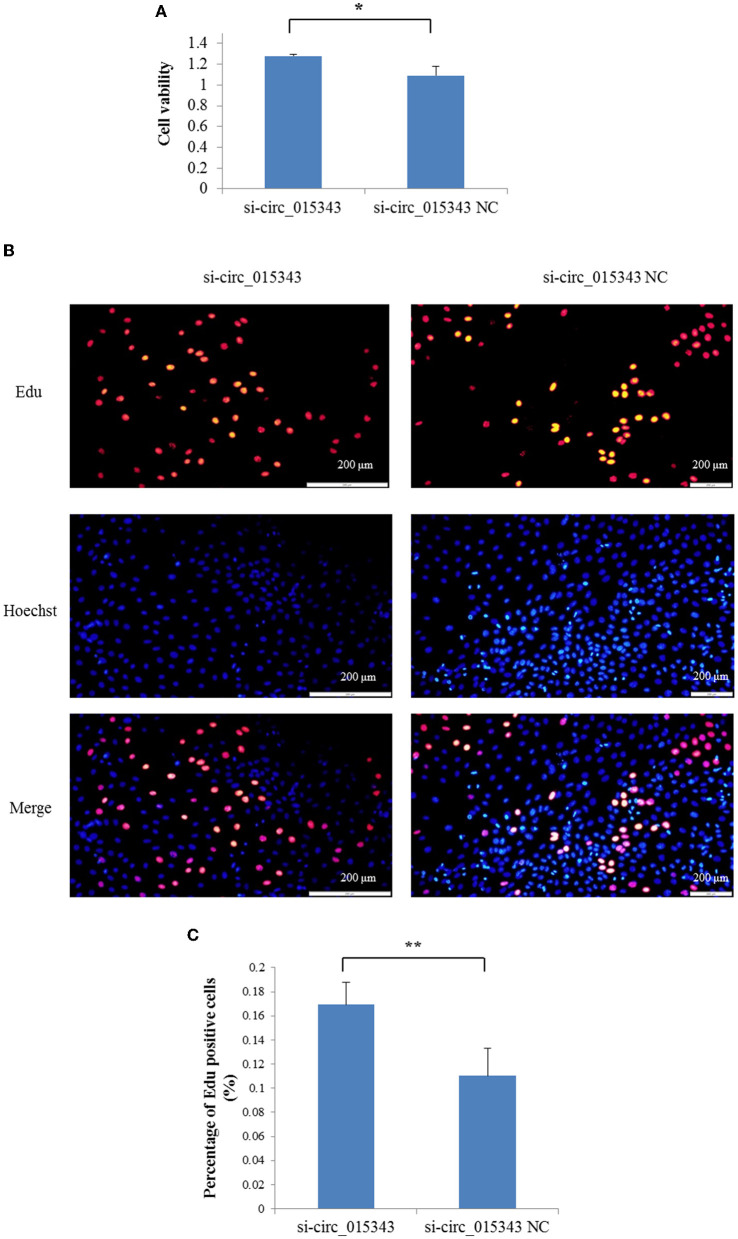
The effect of circ_015343 on the viability and proliferation of ovine mammary epithelial cells. **(A)** The viability of ovine mammary epithelial cells detected by using CCK8 assay when si-circ_015343 was transfected into ovine mammary epithelial cells. **(B)** The effect of circ_015343 on the proliferation of ovine mammary epithelial cells was detected by using Edu assay. **(C)** The proportion of Edu-labeled positive mammary epithelial cells in ovine total mammary epithelial cells. **p < 0.01.

**Figure 8:**
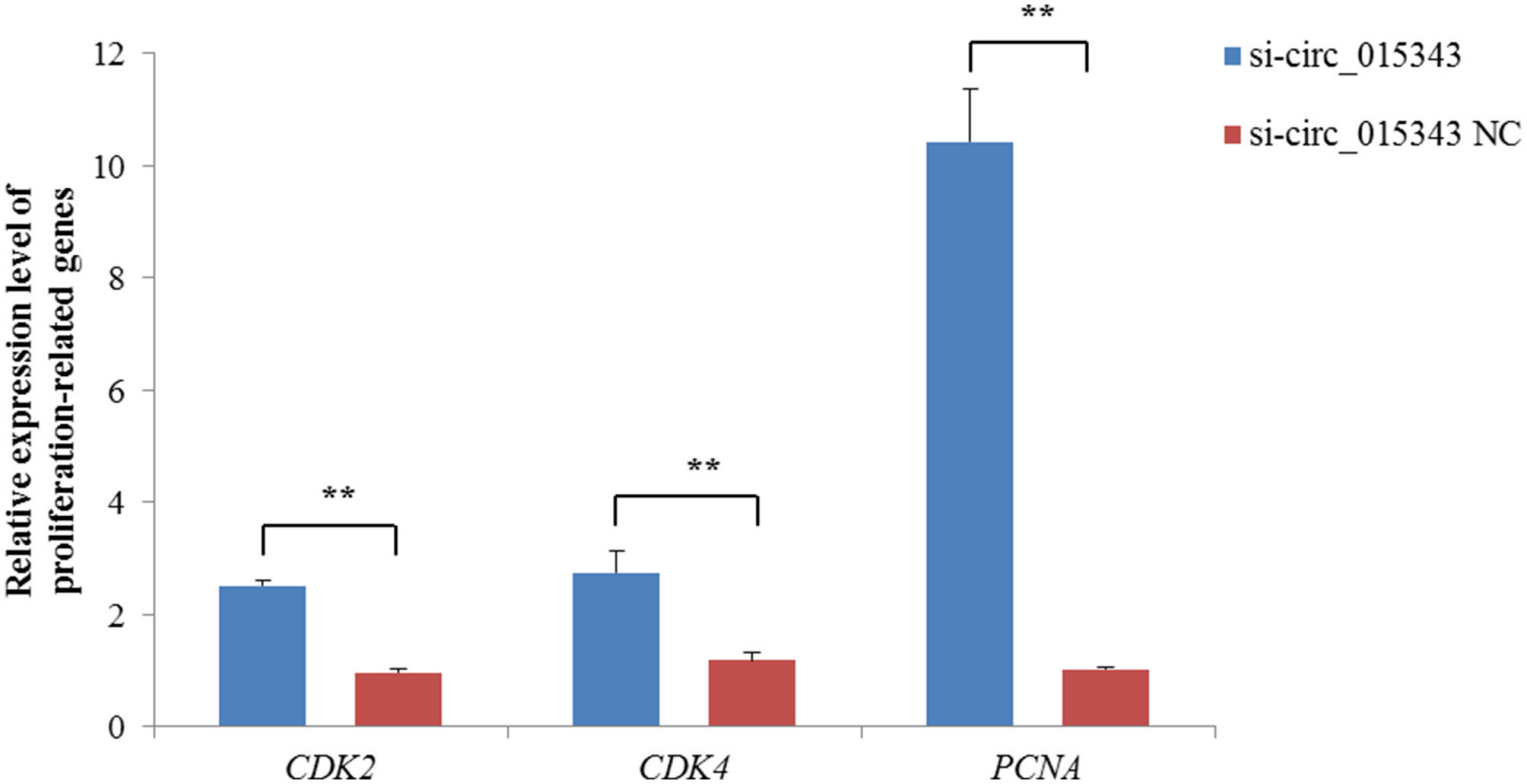
Relative expression levels of proliferation-related genes when si-circ_015343 was transfected into ovine mammary epithelial cells. **p < 0.01.

The CCK8 result showed that si-circ_015343 significantly increased the viability of ovine mammary epithelial cells (*p* = 0.014) (Figure 7A). The Edu assay result revealed that si-circ_015343 increased the number of Edu-labeled proliferated ovine mammary epithelial cells (Figures 7B,C). Meanwhile, si-circ_015343 also increased the expression levels of *CDK2* (*p* = 0.000), *CDK4* (*p* = 0.005) and *PCNA* (*p* = 0.000) when compared to NC group (Figure 8). These suggest that circ_015343 inhibited the viability and proliferation of ovine mammary epithelial cells.

### Construction of a CircRNA-MiRNA-MRNA Regulatory Network

**Figure 9:**
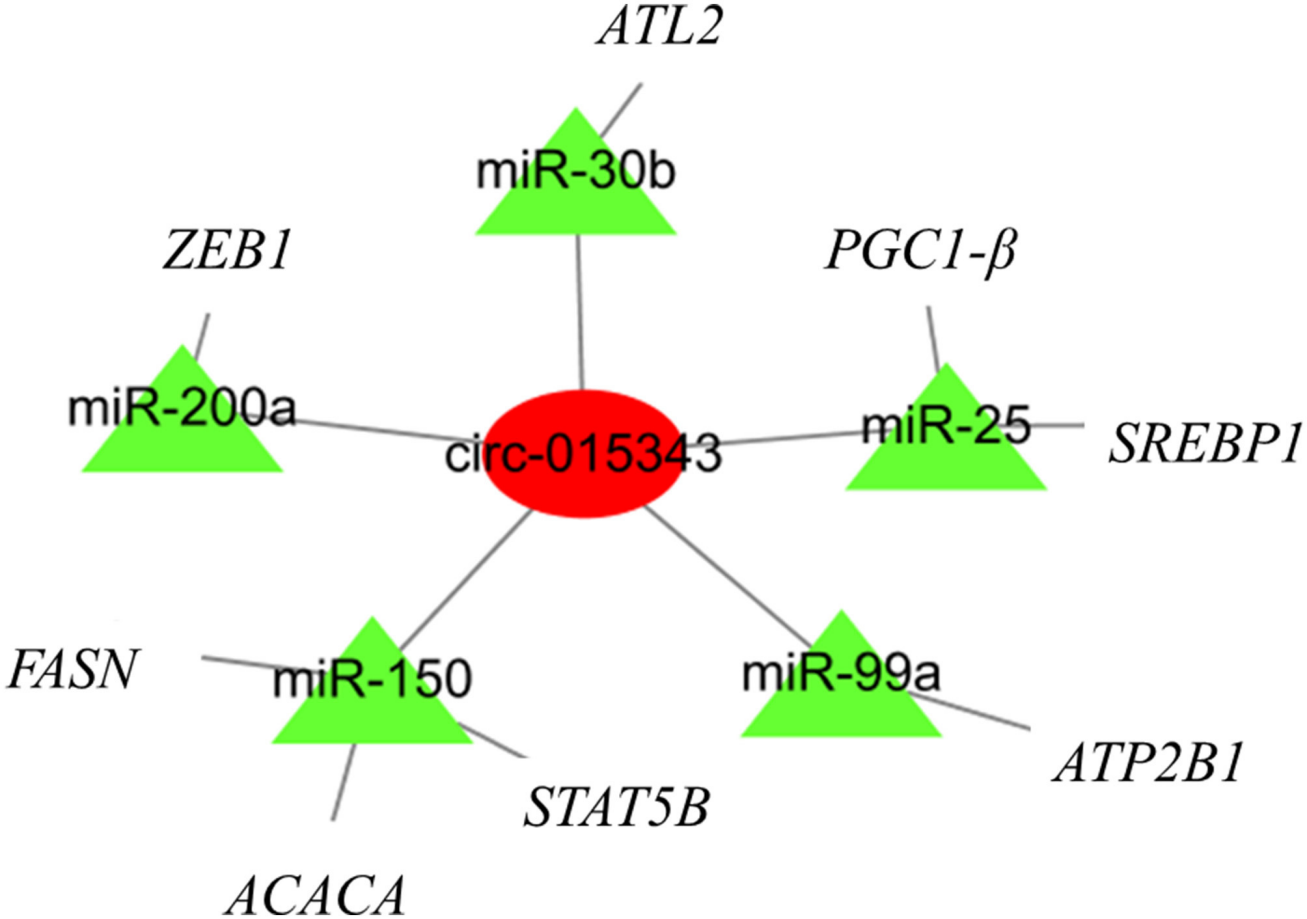
The circRNA-miRNA-mRNA regulatory network. The red circle represents circ_015343, while green triangles indicate predicted miRNAs for circ_015343.

A total of 27 miRNA binding sites were predicted for circ_015343. For clearly presenting the interaction effect of circ_015343 and miRNAs, five miRNAs (miR-200a, miR-30b, miR-150, miR-99a, and miR-25) were further selected as these miRNAs have been reported to be associated with mammary gland development and lactation (18–22). Although there were 2,774 target genes for the five miRNAs, seven target genes were selected as their target relationship with these miRNAs has been reported (18–22). A circRNA-miRNA-mRNA interaction network was finally constructed (Figure 9).

## Discussion

This study constructed the tissue expression profiles of circ_015343, and also investigated the effect of inhibited circ_015343 on ovine mammary epithelial cells, as well as the expression levels of milk fat synthesis and proliferation marker genes. A growing body of research shows that circRNAs are involved in mammary gland development and lactation in mammals. For example, circ_006258 promoted proliferation and milk synthesis of goat mammary epithelial cells (23). The circ_140 inhibited casein secretion and lipid formation in goat mammary epithelial cells (24). The circ_11103 increased the contents of triglycerides and unsaturated fatty acids in bovine mammary epithelial cells (25). In this study, we first confirmed the authenticity of circ_015343 by using RT-PCR and Sanger sequencing. The methods are usually used to validate the presence of circRNAs (26). The circ_015343 originated from *AASS*. *AASS* encodes a bifunctional enzyme that catalyzes the first two steps of the mammalian lysine degradation pathway, resulting in the conversion of lysine to α-aminoadipic semialdehyde (27). The inhibition or modification of *AASS* reduced the degradation of lysine (28). Lysine is the main essential amino acid for milk protein synthesis, suggesting that the protein is of great significance in regulating the synthesis of milk components. For example, the mammary gland tissue of dairy cows used 86% of mammary lysine to synthesize milk protein (29). In this context, circ_015343 produced by *AASS* may play important role in the synthesis of milk protein.

It was notable that circ_015343 was an annot_exon circRNA. It has been reported that annot_exons were the most common circRNAs in the mammary gland and other tissues (5, 30). The type of annot_exons circRNA is mainly located in the cytoplasm (31) and may therefore act as miRNA sponges to relieve the inhibition of the target mRNAs by miRNAs (32). The subsequent RT-qPCR results by using RNA extracted from the nucleus and cytoplasm of ovine mammary epithelial cells also confirmed that as an annot_exon circRNA, circ_015343 is mainly located in the cytoplasm of ovine mammary epithelial cells. It is therefore inferred that circ_015343 plays its biological function more in the cytoplasm.

The circ_015343 and *AASS* were found to be expressed in ovine eight different tissues, with the highest expression level in the mammary gland. This likely reflects the tissue-specific expression of circ_015343. In this respect, differential tissue-specific expression of circRNAs has also been reported in humans (33). The wide expression of circ_015343 in different tissues suggests that the circRNA may play pleiotropic roles in a variety of biological processes. However, the highest expression of circ_015343 indicates its main biological function in the regulation of mammary gland development.

It is noteworthy that circ_015343 had lower expression in the mammary gland tissue of Small Tail Han ewes at peak lactation. Given that the Small Tail Han ewes had higher milk yield, milk fat rate, and milk protein rate compared to Gansu Alpine Merino ewes (5), it is therefore inferred that circ_015343 may inhibit the lactation and synthesis of milk fat and protein in sheep. The speculation was confirmed by subsequent research into the effect of circ_015343 on ovine mammary epithelial cells.

In the study, si-circ_015343 significantly increased the viability and the Edu-labeled positive number of ovine mammary epithelial cells. Meanwhile, si-circ_015343 also increased the expression levels of proliferation genes *CDK2, CDK4*, and *PCNA*. The proteins CDK2 and CDK4 are cell cycle regulators (34). PCNA occurs as a component of multiprotein complexes during cell proliferation, which plays an essential role in DNA replication (35). The three genes are positively correlated with cell proliferation. The result from RT-qPCR was therefore consistent with the observation obtained from the Edu assay. These suggest that circ_015343 inhibited the viability and proliferation of ovine mammary epithelial cells. It is well known that the number and viability of mammary epithelial cells are positive correlation with the ability of the mammary gland to secrete milk and the contents of fat and protein in milk (36). This together with the lower expression level of circ_015343 in the mammary gland of Small Tail Han ewes with higher milk yield, milk fat, and protein contents, suggests that circ_015343 inhibited the lactation and the synthesis of milk fat and milk protein by inhibiting the proliferation and viability of ovine mammary epithelial cells in sheep. Meanwhile, si-circ_015343 decreased the expression level of its parent gene *AASS*, suggesting that circ_015343 cis-regulated *AASS* in expression. The cis-regulation relationship of specific circRNA with its parent gene has been reported for circEIF3J and circPAIP2 (37).

In the study, the inhibition of circ_015343 increased the expression levels of milk fat synthesis marker genes *FABP4, ACACA*, and *SREBP1*. This result suggests that circ_015343 inhibited the expression of these genes. FABP4 protein was found to improve milk yield, protein content in milk (38), and content of medium and long chain fatty acids in milk (39). *ACACA* is involved in milk fatty acid *de novo* synthesis (40) and is positively correlated with milk fat yield (41). *SREBP1* promoted the synthesis and secretion of milk fat in mammary epithelial cells of dairy cows (42). The three milk fat synthesis marker genes are involved in the AMPK signaling pathway and PPAR signaling pathway that are associated with milk fat synthesis (43). The inhibition effect of circ_015343 on the synthesis of milk fat was also in accordance with our findings obtained from CCK8 and EDU assays, as well as its differential expression between Small Tail Han ewes and Gansu Alpine Merino ewes.

A circRNA-miRNA-mRNA regulatory network found that circ_015343 would bind miRNAs to regulate the expression of functional genes related to mammary gland development and lactation. For example, PPARG Coactivator 1 Beta (*PGC-1β*) has been shown to stimulate the expression of genes involved in lipid metabolism (44). Fatty acid synthase (*FASN*) is a crucial enzyme of fatty acid *de novo* synthesis in the mammary gland and has been proved as the main source of short and medium-chain fatty acids in milk (45). The analysis illustrates again the regulatory role of circ_015343 in milk synthesis and mammary gland development in sheep.

## Conclusion

This study describes tissue-specific expression of circ_015343 and its inhibited effect on lactation and contents of fat and protein in milk. Our results provide a better understanding of the roles of circ_015343 in mammary gland development and lactation in sheep.

## Data Availability Statement

The raw data supporting the conclusions of this article will be made available by the authors, without undue reservation.

## Ethics Statement

The animal study was reviewed and approved by Animal Experiment Ethics Committee of Gansu Agricultural University (Lanzhou, China).

## Author Contributions

XW and JW did the data analysis and wrote the manuscript. HZ, YLi, LL, YLu, XL, SL, ZH, and ML collected the samples. LH and LQ analyzed the data. JW did the project administration and revised the manuscript. All authors contributed to the article and approved the submitted version.

## Funding

This work was financially supported by the National Natural Science Foundation of China (32060746 and 31860635), the Yong Supervisor Support Fund of Gansu Agricultural University (GAU-QDFC-2020-01), and the Fuxi Young Talents Fund of Gansu Agricultural University (Gaufx-02Y02).

## Conflict of Interest

The authors declare that the research was conducted in the absence of any commercial or financial relationships that could be construed as a potential conflict of interest.

## Publisher's Note

All claims expressed in this article are solely those of the authors and do not necessarily represent those of their affiliated organizations, or those of the publisher, the editors and the reviewers. Any product that may be evaluated in this article, or claim that may be made by its manufacturer, is not guaranteed or endorsed by the publisher.
